# Syzygium Jambolanum (Jambol) in Diabetes*The fruit of Syzygium Jambolanum, natural order Myrtaceæ, indigenous in the East Indies.The fruit is fusiform, about fifteen (15) milimetres long, ten (10) milimetres thick, very hard, brownish-black, astringent, aromatic. In powder it reminds one of pepper in odor. It has been recommended for the elimination of sugar from the urine.—*The Pharmacist.*

**Published:** 1885-08

**Authors:** C. E. Clacius

**Affiliations:** Chicago


### SYZYGIUM JAMBOLANUM, (JAMBOL.)*

*By* C. E. CLACIUS, M. D., *Chicago.*

A new drug was mentioned in the *London Lancet* some moths ago under the above name, the bark and seeds of which were recommended as a powerful remedy for diabetes mellitus. It was called a stomachic, carminative, diuretic and astringent. The powdered fruit-stones to be taken in five-grain doses three or four times a day.

From an acquaintance, a diabetic, for whom the writer, during several years, had analyzed the urine and invariably had found from eight to twelve per centum of sugar, the writer received a few weeks ago a portion of urine for examination, with the information that the patient had, for a week before,

*The fruit of Syzygium Jambolanum, natural order Myrtaceæ, indigenous in the East Indies.

The fruit is fusiform, about fifteen (15) milimetres long, ten (10) milimetres thick, very hard, brownish-black, astringent, aromatic. In powder it reminds one of pepper in odor. It has been recommended for the elimination of sugar from the urine.—*The Pharmacist.*

used a new remedy, an Indian plant, with such remarkable effect, that he wished to have his curiosity, about the quantity of sugar, gratified.

The urine showed a natural color and smell, had a specific gravity of 1036, and contained no sugar. A few days later the gentleman called in person, and brought with him a small quantity of bark and seeds, which he had obtained directly from London, through the *Lancet*, and reported that during eight days he had taken a decoction of the seeds, corresponding to about half an ounce. Before commencing to take it, his urine had the specific gravity of 1040 to 1042, and he was troubled with a constant dull headache in the occiput. He had to urinate from three to four times a night. The quantity of urine voided was six to eight pints in twenty-four hours. After a week's use of the remedy his headache had disappeared; his sleep was undisturbed, and the quantity of his urine had decreased thirty per centum, and there was no trace of sugar in it. The high specific gravity was caused by a concentration of urea, urates and phosphates in the lessened quantity of fluid. Further observation about the permanency of this favorable change could not be made, as the gentleman left for a European tour a few days later, but he was so elated that he requested the writer to experiment with the drug which he brought, on other patients with the same disease. This has been done, and the result is herewith submitted to the profession for judgment and further investigation:

No. 1. Gentleman, aged seventy-one, suffered from disease since 1880, to his knowledge; then, increasing debility, thirst and flattening of the eyes, more than the large quantity of urine voided, induced him to seek medical aid. The skimmed milk diet, strictly followed for three months, removed the alarming symptoms; after that time he added so-called gluten bread, meat and claret, and felt quite comfortable, although he voided from three to seven pints of water in twenty-four hours, which varied in specific gravity between 1030 and 1040, and in sugar, between eight and twelve per centum. The writer made occasional analyses through all those years. To this patient thirty-two powders were given, each containing five grains of the powdered seed.

The analysis made before he commenced on them showed specific gravity of 1036; sugar, eight per centum. Eight days later, after having taken four powders each day, there was a decrease of twenty-five per centum in the quantity of urine, and in this decreased quantity the sugar had decreased from eight to six per centum, and he was not disturbed during the night. After another week, the same dose of the drug caused another reduction of ten per centum in the quantity of urine, and of sugar down to four per centum, and caused, besides, an inexplicable invigoration of the whole system, so that he ventured to, and was able to drive a spirited horse, which he would not have thought of attempting to do two weeks previous to that time. After a third week, the quantity of urine amounted to from thirty-five to fifty per centum of that passed before the medicine was taken, and the sugar in it to only three per centum.

No. 2. Gentleman, aet 47. Had diabetes for four years, but never very bad. He does not know how much sugar his urine contained during those years. He voided from five to seven pints in twenty-four hours, of a gravity of 1036. He suffered more from the inconvenience of keeping up a diabetic diet than from the symptoms of the disease, excepting the necessity of having to urinate two or three times during night. Before he commenced taking the drug, the gravity was 1036, the amount of sugar was six per centum. After three weeks, the specific gravity was reduced to 1030; the amount of sugar to three per centum ; the quantity of urine to four pints, and for the first time in many years he passed a night without urinating. This patient had partaken of potatoes and sweetmeats during the latter two weeks. He likewise reports a marked improvement in his general feeling.

No. 3. A physician, in the prime of life, not a bad case of diabetes, of the intermittent kind. The urine passed during and after the hours of rest contained sugar amounting to four per centum, while that passed during and after the hours of bodily and mental activity and strain was free from sugar. The effect of the drug on this patient was the same as on the others ; the quantity of urine, its specific gravity and its percentage of sugar were constantly reduced. At one time he thought he had to reduce the daily dose of twenty grains of the drug to ten grains, for fear of reducing the quantity of urine too much.

The limited amount of the drug on hand permitted of no further experiments, but the uniformly favorable results induced the writer, several weeks ago, to send to London for a supply, which is now due and daily expected. He would have waited for its arrival, and perhaps investigated a little more, before communicating this limited experience, but the fact that he is soon to remove to and permanently locate at Los Angeles, California, prompts him to bring these limited experiments and their results to the knowledge of the medical profession, and to ask the members of it to continue the investigation, whenever an opportunity is offered.

The action of the drug seems to be direct on the nerve-centers, and if such is the case, its beneficial effects is probably not limited to diabetes mellitus. The seed and bark of the plant Syzygium Jambolanum were recommended. The writer had a sample of each ; of the bark he made a tincture, tried it, but found its efficiency almost null. The seed, or fruit-stones, appear to be of a gum-resinous nature, and as the dose is small and the taste not disagreeable, he gave the powdered seed; the quantity on hand being too small to justify risky experiments of extracting them with various menstrua. When freshly pulverized, the seeds issue a delicate pepperlike flavor, to preserve which it may be advisable to dispense the powder in gelatine capsules.

The drug will be kept, after its arrival, at the store of Messrs. Vaughn & Sawyer, Apothecaries, 322 West Madison Street, Chicago.

